# Sex-Specific Differences in the Pathophysiology of Hypertension

**DOI:** 10.3390/biom15010143

**Published:** 2025-01-18

**Authors:** Hannah Zhang, Pawan K. Singal, Amir Ravandi, Inna Rabinovich-Nikitin

**Affiliations:** 1 Department of Physiology and Pathophysiology, St. Boniface Hospital Albrechtsen Research Centre, Institute of Cardiovascular Sciences, Rady College of Medicine, Max Rady Faculty of Health Sciences, University of Manitoba, Rm. 3042, 351 Taché Avenue, Winnipeg, MB R2H 2A6, Canada; zhangh22@myumanitoba.ca (H.Z.); psingal@sbrc.ca (P.K.S.); amir.ravandi@umanitoba.ca (A.R.); 2 Department of Pharmacology and Therapeutics, St. Boniface Hospital Albrechtsen Research Centre, Institute of Cardiovascular Sciences, Rady College of Medicine, Max Rady Faculty of Health Sciences, University of Manitoba, Rm. 3042, 351 Taché Avenue, Winnipeg, MB R2H 2A6, Canada; 3 Section of Cardiology, Department of Medicine, Rady College of Medicine, Max Rady Faculty of Health Sciences, University of Manitoba, Winnipeg, MB R2H 2A6, Canada

**Keywords:** hypertension, fat metabolism, women, sex hormones

## Abstract

Hypertension is one of the most common comorbidities in cardiometabolic diseases, affecting nearly one third of adults. As a result, its pathophysiological mechanisms have been studied extensively and are focused around pressure natriuresis, the renin–angiotensin system (RAS), the sympathetic nervous system, oxidative stress, and endothelial dysfunction. Additionally, hypertension secondary to other underlying etiologies also exists. While clinical evidence has clearly shown differences in hypertension development in males and females, relatively little is known about the pathophysiological mechanisms behind these differences. Sex hormones likely play a key role, as they modulate many factors related to hypertension development. In this review, we postulate the potential role for sexually dimorphic fat metabolism in the physiology of hypertension. In brief, estrogen promotes subcutaneous fat deposition over visceral fat and increases in mass via adaptive hyperplasia rather than pathogenic hypertrophy. This adipose tissue subsequently produces anti-inflammatory effects and inhibits metabolic dysfunction-associated fatty liver disease (MAFLD) and RAS activation, ultimately leading to decreased levels of hypertension in pre-menopausal females. On the other hand, androgens and the lack of estrogens promote visceral and ectopic fat deposition, including in the liver, and lead to increased circulating pro-inflammatory cytokines and potentially subsequent RAS activation and hypertension development in males and post-menopausal females. Understanding the sex-specific differences in fat metabolism may provide deeper insights into the patho-mechanisms associated with hypertension and lead to more comprehensive sex-specific care.

## 1. Introduction

Hypertension is a growing health issue in the aging population, affecting nearly one third of adults globally [1]. It is defined by a persistent systolic blood pressure (SBP) ≥ 140 mmHg and/or a diastolic blood pressure (DBP) ≥ 90 mmHg. Hypertension is the most common modifiable risk factor contributing to a number of major causes of mortality, including cardiovascular disease, heart failure, kidney failure, and vascular dementia [2]. Risk factors for developing hypertension include lifestyle habits, such as low physical activity and poor diet (particularly high sodium intake), as well as high cholesterol, high body mass index, the presence of diabetes and/or chronic kidney disease, older age, and lower socioeconomic status [3,4]. Despite its overwhelming burden on the global healthcare system, hypertension is often not managed appropriately, with only 37% of hypertensive patients in the United States having their blood pressure well controlled [2]. While this low rate is, in part, due to clinical shortcomings, there are still significant advances to be made in understanding the pathophysiology and heterogeneity of this disease.

Hypertension is typically classified into two major categories: primary hypertension and secondary hypertension. Primary hypertension, also known as essential hypertension, is considered idiopathic and makes up the majority of hypertensive patients. Secondary hypertension affects 5–10% of patients and is developed secondary to an underlying pathology, which causes elevated blood pressure. The most common etiologies for secondary hypertension vary by age and include renovascular hypertension, hormonal imbalances, and anatomic abnormalities. Certain clinical indices can lead healthcare workers to investigate these causes, such as persistent and extreme hypertension despite treatment, preadolescent onset of hypertension, pregnancy-related hypertension, and sudden rises in blood pressure in previously stable patients [5].

**Figure 1:**
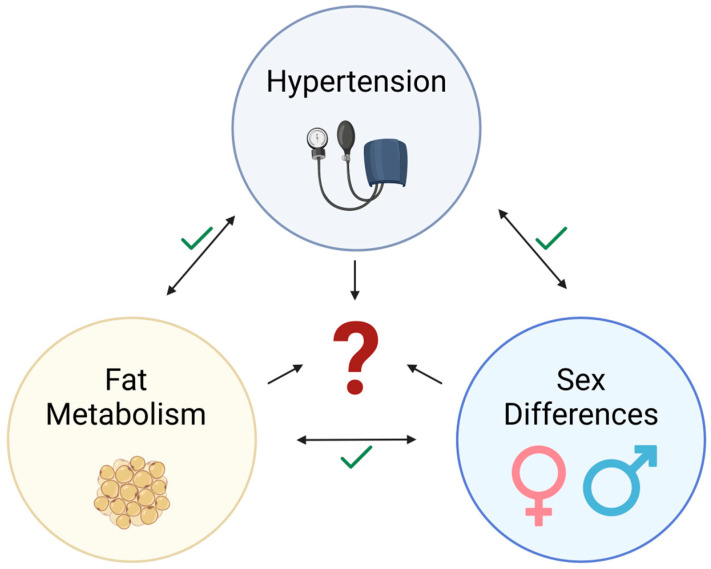
The intersection of hypertension, fat metabolism, and sex differences as an emerging area of research.

Despite the high prevalence of hypertension, there are still several knowledge gaps in our understanding of the pathology of high blood pressure, such as identifying predisposing risk factors to hypertension that can aid in patient risk assessments, prevention, and targeted therapy development, as well as understanding the complexities of the role of other physiological systems in hypertension development. In this review, we will briefly discuss the major causes and mechanisms of primary and secondary hypertension and will highlight the sex-specific differences in hypertension development, which have historically been overlooked. Finally, we will focus on the potential role of fat metabolism in sex differences in the pathophysiology of hypertension, as a new emerging area of research (Figure 1).

## 2. Main Pathophysiological Pathways in Primary Hypertension

### 2.1. Kidney Control of Water Retention

The kidneys are vital in maintaining long-term salt and water balance in the body and preventing circulatory collapse. In normal healthy conditions, a rise in blood pressure will cause the kidneys to increase sodium and water excretion via pressure natriuresis and diuresis to preserve fluid balance [6]. Chronic overload of dietary sodium is met with compensatory water reabsorption in the kidneys to maintain electrolyte balance. Since the industrial revolution, sodium consumption has increased dramatically and is strongly correlated with increased average blood pressures. Importantly, in primary hypertension, there are rarely identifiable kidney abnormalities except that pressure natriuresis appears altered. Instead of increased natriuresis and diuresis in response to chronically elevated pressures, it has been observed that the rate of sodium excretion remains normal and unchanged, suggesting an altered setpoint in hypertensive patients and, thus, a lack of stimulation of compensatory mechanisms [6]. Previous studies have also postulated a sexually dimorphic distribution of kidney transporters in both rodent models and in humans [7,8,9,10]. Specifically, female rat nephrons have lower Na^+^ and water transporter activities along their proximal tubules but a higher abundance of Na^+^ transporters in the distal nephron, ultimately resulting in no significant functional difference in urine excretion between the sexes [7,9]. While these differences are thought to provide females a renal reserve capacity for water and salt preservation in pregnancy and lactation [8], it is unlikely that this is a major mechanism in the sex differences in hypertension.

### 2.2. The Renin–Angiotensin System (RAS)

**Figure 2:**
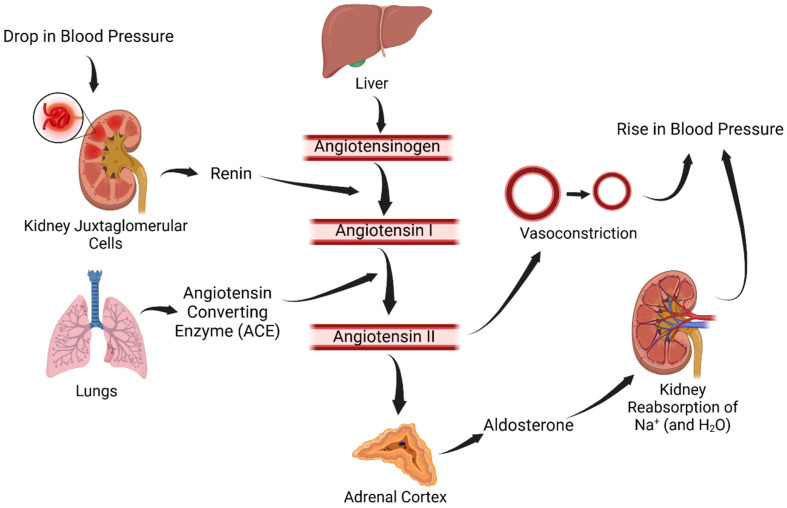
The renin–angiotensin system.

The primary method by which the kidneys exert their control on the vasculature is through the RAS, summarized in Figure 2. Specifically, drops in blood pressure will cause the juxtaglomerular cells in the kidney to activate and cleave prorenin to renin. Renin will then cleave circulating angiotensinogen, which is made continuously by the liver, into angiotensin I. Angiotensin I is then cleaved by an angiotensin-converting enzyme (ACE) into angiotensin II, which is biologically active. Angiotensin II exerts its effects through two main receptor types spread across multiple organs: type I (AT1) and type II (AT2). In the kidney, angiotensin II increases sodium reabsorption by increasing the Na-H exchange at the proximal convoluted tubule and causes fluid shift into the vasculature due to the increased osmolarity of blood. Angiotensin II also stimulates aldosterone secretion from the adrenal gland, which promotes sodium and water retention and potassium excretion by the kidneys, further increasing the intravascular fluid volume. The sympathetic system is activated by angiotensin II, leading to vasoconstriction. Both clinical and experimental studies have provided evidence that high RAS is implicated in essential hypertension development [11]. It has also been well established that sex differences exist in the regulation and effects of RAS [12,13,14,15]. In fact, the RAS activity is increased with androgens and antagonized by estrogens [14], which will be discussed in more detail below. Furthermore, the primary action of angiotensin II on the proximal tubule is likely a reason why ACE inhibitors have a more potent blood pressure-lowering effect in males compared to females [8,16,17,18]. Therefore, the RAS is a key target for blood pressure modulation and has important implications in the sex differences seen in hypertension.

### 2.3. Neural Control and Sympathetic Signaling

The brain has significant control over blood pressure regulation through sympathetic signaling and baroreceptor reflex modulation. Early studies caused some speculation as to the significance of the sympathetic system in the long-term regulation of blood pressure, but more recent studies have shown the importance of autonomic control [19]. Specifically, the baroreceptor reflex, which is regulated by the carotid nerve and the central nervous system (CNS), can fail from denervation or CNS disorders. These patients have higher resting blood pressures due to deregulated homeostatic pathways and have a higher baseline sympathetic activity. It has also been suggested that the role of the sympathetic nervous system in resting blood pressure control is age- and sex-dependent [19], which provides an important clue on the role of sex hormones in hypertension pathogenesis and will be discussed in more detail below.

### 2.4. Oxidative Stress and Inflammation

Endothelial oxidative stress and inflammation have been implicated in the pathogenesis of hypertension, although the mechanisms linking these conditions have not yet been fully elucidated [20,21]. Reactive oxygen species (ROS) are important signaling molecules in healthy tissues and are normally balanced with antioxidant pathways; however, ROS accumulation can occur when these pathways are imbalanced, leading to oxidative stress. ROS can interact with the immune system by activating a myriad of immune cells, which leads to inflammation and organ injury. Specifically in the context of hypertension, endothelial, neural, and renal tissues are adversely affected. Furthermore, angiotensin II (AngII) has been shown to cause oxidative stress in physiological and supraphysiological doses by increasing vascular superoxide levels via NADPH oxidase [22,23]. The spontaneously hypertensive rat (SHR) model has given several insights into the role of oxidative stress in hypertension and sex differences, including higher levels of oxidative stress in male SHRs than in females and higher plasma superoxide dismutase activity in females [24,25]. More research is needed to fully understand the cell types and mechanisms involved in the complex relationships between oxidative stress, inflammation, and hypertension.

### 2.5. Endothelial Dysfunction

While the endothelium was previously thought to play a relatively passive role as a physical barrier, it is now clear that it plays an important bioactive role in the pathophysiology of many diseases, with a particularly critical role in cardiovascular diseases. Endothelial dysfunction, characterized by an altered vasodilatory ability and aberrant activation, is another potential component in hypertension development. While a healthy endothelium has vasodilatory properties that are athero-protective, as well as anticoagulant and profibrinolytic characteristics, a dysfunctional endothelium lacks the capacity to perform many of these important regulatory roles. Nitric oxide (NO), the most potent endogenous vasodilator, has an altered metabolism when faced with oxidative stress, and the decreased NO bioavailability leads to platelet aggregation, vasoconstriction, vascular smooth muscle hypertrophy, and atherosclerosis [26]. However, the experimental evidence on the role of decreased NO synthesis being involved in systemic hypertension has been controversial, as there seems to be heterogeneity in the effect of endothelium-related vascular relaxation [26]. Although it has been shown that NO production is better preserved in females compared to males, its role in the sexual dimorphism of hypertension has not been well characterized [27]. Overall, there are intricate interactions between endothelial function, inflammation, and oxidative stress in hypertension development, and the components of these pathways could serve as potential therapeutic targets in the future.

## 3. Common Causes of Secondary Hypertension

### 3.1. Renovascular Hypertension

Renovascular hypertension is a potentially reversible cause of secondary hypertension and primarily affects patients with atherosclerosis in the renal artery, termed renal artery stenosis, but can also be due to fibromuscular dysplasia [5]. Goldblatt et al. were the first to describe a potential mechanism in the 1940s by showing that preglomerular constriction of the renal artery leads to insufficient blood flow to the kidney and, thus, increased renin secretion, which activates the renin–angiotensin system (RAS) [28].

### 3.2. Endocrine-Related Hypertension

The endocrine system has a profound effect on blood pressure regulation, and, thus, hormonal abnormalities can cause persistently high blood pressure. Primary hyperaldosteronism is a prevalent cause of secondary hypertension and is characterized by the excessive production of the aldosterone hormone in the adrenal glands, independent of RAS activity [5]. The main role of aldosterone in the body is to increase water reabsorption in the kidneys by increasing sodium re-uptake; however, excess aldosterone levels lead to increased intravascular volumes and elevated blood pressures. Cushing’s syndrome is another hormonal cause of secondary hypertension, caused by high cortisol levels. Sustained excessive cortisol levels lead to excessive salt reabsorption and increased vascular sensitivity to angiotensin II and catecholamines, all of which contribute to hypertension [29].

Thyroid disorders can also lead to the development of hypertension. In hyperthyroidism, high levels of biologically active thyroid hormone (T3) can cause decreased systemic vascular resistance and activation of RAS. T3 also stimulates cardiac contractility and increases cardiac output via gene regulation. Together, these elements cause patients with hyperthyroidism to develop hypertension with a wide pulse pressure. Hypothyroidism can lead to diastolic hypertension and a narrowed pulse pressure, slowing the relaxation phase of the cardiac cycle and increasing susceptibility to atherosclerosis peripherally [30].

Pheochromocytomas are tumors in the chromaffin cells of the adrenal gland and are a rare cause of secondary hypertension by synthesizing excess catecholamines. The excess of catecholamines acts on α_1_-adrenergic receptors in the vascular smooth muscle cells to cause vasoconstriction and β_1_-adrenergic receptors, which have positive inotropic effects and stimulate renin release [31]. Together, these mechanisms cause a rise in mean systemic blood pressure. Overall, the cardiovascular system is very sensitive and receptive to hormonal changes in the body, and, therefore, endocrine disturbances are a significant cause of secondary hypertension.

### 3.3. Pregnancy-Related Hypertension

Two of the most common complications during pregnancy are gestational hypertension and pre-eclampsia and, in some cases, subsequent chronic hypertension [32]. Pre-eclampsia, in particular, carries significant morbidity and mortality risks and is the leading cause of maternal death [33]. The core of pre-eclampsia involves placental dysfunction and hypoxia, which implicates oxidative stress, inflammation, and genetic and environmental factors [33]. There are also higher levels of the antiangiogenic protein sFLT1 in pre-eclamptic women, which leads to endothelial dysfunction and impaired relaxation [33]. The presence of these placental abnormalities early in the pregnancy will lead to systemic vascular dysfunction in the later trimesters, which can result in proteinuria and hypertension. There have also been studies that showed increased AngII sensitivity during and before the onset of pre-eclampsia, despite lower levels of circulating renin and AngII compared to normal pregnancy [34,35]. However, more studies are needed to understand the molecular mechanisms at play to either prevent its onset or improve the treatments for patients with gestational hypertension and pre-eclampsia.

### 3.4. Other Common Causes of Hypertension

Anatomic anomalies can also lead to hypertension. Specifically, patients with coarctation of the aorta classically have chronic hypertension that persists even with surgical repair. Obstructive sleep apnea (OSA), which has both modifiable and anatomic causes, is also a common reversible risk factor for hypertension. The hypoxia experienced in OSA can lead to inflammation and oxidative stress, as well as interactions with the sympathetic nervous system [36], which can lead to elevated blood pressures.

## 4. Role of Sex in Hypertension

Traditionally, cardiovascular and cardiometabolic diseases were thought to primarily affect males, leading to the belief that females are “protected” from these conditions. Additionally, the consistent hormone cycling in females during menarche followed by hormonal changes in pregnancy and menopause makes the female physiology more complex and difficult to study in preclinical models, which also contributed to the historical exclusion of women from clinical trials [37]. In the case of hypertension, the prevalence is higher in men until the age of 60, at which point females undergo menopause and have rapidly inclining rates of hypertension that surpass men [38]. This indicates that sex hormones may play an important role in the pathophysiology of hypertension. Although it is now evident that both sexes are at risk of developing hypertension [39,40], there is still a significant knowledge gap in the understanding of sex-specific mechanisms and risk factors for hypertension. It is well established that a lower blood pressure threshold in females confers the same cardiovascular disease risk compared to a higher blood pressure threshold in their male counterparts [38,39], yet clinically the same thresholds are still used in both sexes. This is further complicated by the fact that many studies have shown sex differences in the response to different antihypertensive pharmacotherapies [41]. Although there have been several preclinical and clinical observations of sex differences in physiological blood pressure control, such as differences in salt sensitivity and renal excretion [42,43,44], the mechanisms are still unclear [45]. It has generally been postulated that changes in sex hormones are responsible for the differences in the pattern of hypertension development [6]. Research on post-menopausal hypertension has implicated the role of oxidative stress, endothelin, and RAS, as well as sympathetic nervous system activation secondary to obesity [46]. This section will explore what is known about the sex differences in physiology in relation to hypertension and identify an area that warrants further investigation.

### 4.1. Role of Estrogen and Progesterone Signaling

Estrogens have long been thought to have cardioprotective effects, mostly based on findings from preclinical animal studies [40]. Since diminishing endogenous estrogen levels in menopausal women coincides with the increasing incidence of hypertension, many have speculated on its critical role in the pathophysiology of hypertension. Furthermore, some studies have shown that women who undergo hysterectomy or oophorectomy, which results in the sudden loss of endogenous estrogen, are at an increased risk of hypertension [47,48]. However, research in hormone replacement therapy has led to doubts being cast on this theory, as exogenous estrogen supplementation in post-menopausal women does not reverse the risk of developing CVD [49], although it does decrease renin and increase angiotensinogen levels [50]. It has been shown that through its binding to its three types of receptors, estrogen can cause vasodilation, have anti-inflammatory and antioxidant effects, alter gene expression, and reduce cholesterol deposition on arterial walls [49,51]. Furthermore, estrogen’s interaction with the protective angiotensin (1–7) may also confer cardioprotective effects via the attenuation of RAS activation [52]. Estrogen is also important in regulating the distribution of white adipose tissue, favoring subcutaneous fat deposition over visceral fat deposition [53]. This point is important as subcutaneous fat is considered cardioprotective, while visceral fat is pro-inflammatory, suggesting its link to the pathophysiology of hypertension [54,55,56]. Additionally, estrogen stimulates vasodilatory NO production, although this is not thought to be the primary mechanism by which estrogen lowers blood pressure [22].

While less studied directly than estrogen, progesterone may also have indirect blood pressure modulatory effects. For example, one study showed that progesterone may have moderate impacts on the upper airway dilator muscle activity, which could prevent the development of obstructive sleep apnea and subsequent hypertension [57,58]. However, in a clinical study looking at pregnancy among women undergoing assisted reproduction, progesterone levels did not correlate with the rates of hypertensive diseases, but they did correlate with intrauterine growth restriction [59]. Therefore, it is still unclear if progesterone plays a direct role in hypertension development.

### 4.2. Role of Androgen Signaling

Testosterone and dihydrotestosterone (DHT) are the major androgens in males that contribute to the dimorphic features that are classically associated with the different sexes. Both in animal models and clinical studies, increased androgen levels contribute to chronic blood pressure rises [22]. For example, females with polycystic ovarian syndrome (PCOS), characterized by hyperandrogenism, are at a higher risk of developing hypertension [58]. Some experimental evidence has suggested that testosterone dampens the pressure–natriuresis relationship in the kidneys [22]. Furthermore, androgens are speculated to play a role in upregulating the RAS, as men have higher plasma renin activity compared to women [22]. Experimental evidence has also shown that androgen treatments exacerbate blood pressure elevation through the RAS [22]. One proposed mechanism for this result is that androgens increase AngII levels via RAS, which leads to oxidative stress and renal vasoconstriction, ultimately resulting in increased blood pressures [22].

### 4.3. Role of Chromosomal Differences

In general, every cell in the body has either XX or XY chromosomes, which determines the sex of the individual. These sex chromosomes carry sex-determinant genes and confer sexual dimorphism. It is thought that the Y chromosome is in part responsible for differences between sexes in blood pressure and stress responses [60]. A preclinical study in hypertensive rats showed a localization of genes affecting renal electrolyte excretion on the Y chromosome [61]. The interaction between rat Y chromosome isoforms and RAS has also been studied, but as only one isoform exists in humans, there is perhaps reduced translatability of these studies [62]. Clinical studies, however, have found varying results on genetic variation in the Y chromosome being associated with higher blood pressure [63,64]. More recently, evidence suggests the role of adaptive immunity in Y-chromosome-dependent differences in blood pressure and hypertension risks [62].

### 4.4. Hypothesis—Does Fat Metabolism Lead to Sex Differences in Hypertension?

Research on sex-specific differences in hypertension is in the beginning stages. Therefore, in this final section, we would like to focus the discussion on a potential mechanism of hypertension that relies on differences in fat metabolism between men and women and may warrant further investigation.

It is well established that there are significant sex differences in the development of cardiometabolic diseases, which typically comprise diabetes, fatty liver disease, and cardiovascular disease. As in the case of hypertension, these diseases are often thought to be epidemiologically dominated by males until females reach menopause. Some of the most prominent risk factors in the development of cardiometabolic disease involve fat metabolism, such as obesity and dyslipidemia. In general, ingested fats are absorbed in the gut, shuttled to the liver by chylomicrons, and repackaged into lipoproteins to be delivered to the periphery. Lipids are then stored in adipose tissue, where they can be re-mobilized when needed by other tissues for energy .

On a global scale, obesity consistently tends to affect females more than males [65], although some countries, such as Canada, report a higher prevalence of obesity or overweight among males (69.4%) compared to females (56.7%) in adults over the age of 20 [66]. Interestingly, modernized Western countries have different trends in obesity between sexes compared to the rest of the world, and this is, in part, due to societal factors and social norms. There is also a clearly established link between increased weight gain and increased blood pressures [67]. Visceral fat, in particular, contributes to increased blood pressures in obese individuals [67].

To add another layer of complexity to the relationship between fat metabolism and hypertension, adipose tissue is also known to be a primary source of endogenous estrogen and biologically active adipokines [68], with subcutaneous fat having higher conversion of estrone to estradiol compared to visceral adipose tissue [69]. Adipose tissue was traditionally thought to be a fairly benign organ, primarily for fat storage; however, it is now evident that adipose tissue is important in secreting regulatory factors that affect inflammation, glucose tolerance, and cardiovascular health. In healthy adipose tissue, the adipocytes secrete anti-inflammatory factors, such as adiponectin [70]. As the amount of fat deposition increases, adipocytes can hypertrophy, and once a maximal load is exceeded, they can switch to secreting pro-inflammatory molecules, such as TNFα and IL-6 [68,71]. Therefore, the severity of obesity is linked to the amount of systemic inflammation present, which affects the risk of hypertension development. Adipose tissue also secretes several proteins in the RAS, including angiotensinogen and the angiotensin-converting enzyme, with higher levels expressed in visceral adipose tissues than in subcutaneous adipose tissues [72,73]. Older preclinical studies have shown that adipocyte-derived leptin contributes to obesity-associated hypertension in males via inducing endothelial dysfunction and increasing sympathetic activity [74,75,76]. Mouse studies have also shown that leptin can induce hypertension and endothelial dysfunction via aldosterone pathways rather than the sympathetic system in obese females [77], providing evidence for sexually dimorphic roles of adipose tissue in the context of the pathophysiology of hypertension.

Other organs and physiological systems may also be involved in the development of hypertension. There is a growing drive to understand the role of the gut microbiome in the context of cardiovascular diseases, including hypertension [78]. Animal studies have shown that germ-free rats developed hypertension. Similarly, antibiotic treatment induced hypertension, which further implicates the crucial role of gut microbiota in regulating blood pressure [79]. Given the close link between fat metabolism and the gut microbiome and the recent evidence that sex hormones and their receptors modulate the gut microbiome [80,81,82], the axis of hypertension–gut microbiome–sex differences warrants further investigation to better understand the sexually dimorphic development of hypertension.

The liver is another organ that warrants investigation in the context of hypertension. The liver is one of the most important organs in fat metabolism, as it is a major source of de novo lipogenesis and lipoprotein regulation. Results from high-fat feeding studies in rodents consistently show that males develop liver fat accumulation, while females develop more severe obesity [83,84]. These results suggest that female adipose tissue has superior adaptability to chronic increased fat availability, as subcutaneous adipose is more likely to undergo hyperplasia, while visceral fat more readily undergoes pathologic hypertrophy. Furthermore, visceral, but not subcutaneous, fat is known to increase sympathetic activation, induce renal disease, and increase plasma leptin levels [71,85,86]. When the storage capacity of adipose tissue is exceeded, fat can begin accumulating in other organs, including the liver, and in circulation. The increased fat in male livers, which can range from metabolic dysfunction-associated fatty liver disease (MAFLD) to metabolic dysfunction-associated steatotic liver disease (MASLD) to cirrhosis, leads to an increased inflammatory state in the liver. Furthermore, fatty livers were detected in ~30% of patients who were hypertensive without any other risk factors for metabolic syndromes (including obesity), which is higher than the prevalence of MAFLD in the general population [87].

In parallel with this, other studies have shown that high-fat diet feeding in rodents leads to increased obesity in females but higher blood pressure elevation in males [88]. This pattern indicates that adipose fat deposition and hypertension are not necessarily directly positively correlated. Instead, the ability of females to accumulate healthy adipose before becoming dysfunctional could protect them from developing hypertension early in life. Males being more susceptible to unhealthy adipose tissue buildup and liver dysfunction lead them to have an altered fat metabolism, creating a sustained pro-inflammatory state and, thus, are more at risk of developing sustained high blood pressures.

**Figure 3:**
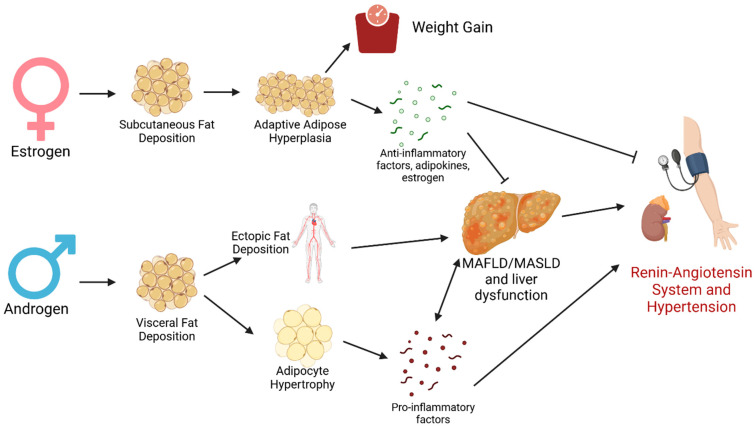
Proposed mechanism of the sex dichotomy in fat metabolism affecting the development of hypertension.

In summary, we propose that fat metabolism plays a central role in the development of hypertension in a sex-dependent manner. With the knowledge that sex hormones play a large role in fat deposition patterns, our hypothesis is visualized in Figure 3 and summarized in the following three points: (1) adipose tissue is a significant source of endogenous estrogen, which acts to reduce RAS activation; (2) healthy adipose tissue has anti-inflammatory properties, which is more common in female subcutaneous fat; and (3) MAFLD alters liver fat metabolism and is more common in males, as well as it may induce systemic inflammation, RAS activation, and sympathetic nervous system stimulation. Together, these mechanisms could lead pre-menopausal females to be at a lower risk of developing hypertension compared to their male counterparts, whereas the significant reduction in systemic estrogen attenuates this protection in post-menopausal females. It is perhaps worthwhile to directly investigate the interaction of fat metabolism, sex, and hypertension, as it could provide insights on disease mechanisms and potentially reduce the disparities in clinical outcomes.

## 5. Conclusions

In any systemic disease research, it is important to realize that no pathways truly work in isolation in the human body. In the case of hypertension, while clinical research has shown significant sex differences, pathophysiological research has yet to fully understand why. In this review, we have proposed the critical role of fat metabolism in the sex differences seen in hypertension. Understanding organ crosstalk may be the key to unlocking new mechanistic insights and clinical targets to improve patient outcomes.

## Data Availability

No new data were created or analyzed in this study.
